# A Comprehensive Review of the Antimicrobial Effects of Hibiscus Species

**DOI:** 10.7759/cureus.73062

**Published:** 2024-11-05

**Authors:** Ahmed E Alharbi, AbdulRahman M AlHussaini, Issam Alshami

**Affiliations:** 1 Medical Laboratories, College of Applied Medical Sciences, Taibah University, Al Madinah, SAU; 2 Basic Medical Sciences, College of Medicine, Taibah University, Al Madinah, SAU

**Keywords:** antibacterial effects, antimicrobial, hibiscus sabdariffa, infection, traditional uses

## Abstract

Hibiscus, particularly *Hibiscus sabdariffa*, is a flowering plant known for its bright red calyxes and diverse uses in traditional medicine. Historically, cultures throughout Africa, Asia, and the Caribbean have utilized hibiscus for its health benefits, including its role in treating high blood pressure, digestive problems, and skin conditions. The rich phytochemical profile of hibiscus, particularly its high content of anthocyanins and flavonoids, has attracted increasing scientific interest, particularly with regard to its antimicrobial properties. Research shows that hibiscus extracts possess significant antimicrobial activities against various pathogens, including bacteria such as *Escherichia coli *and *Staphylococcus aureus* and fungi such as* Candida albicans.* The active ingredients in hibiscus are believed to disrupt microbial membranes, inhibit enzyme activity, and effectively scavenge free radicals, thereby exerting a broad spectrum of antimicrobial effects. The promising antimicrobial properties of hibiscus suggest numerous potential applications, particularly in food preservation, natural antimicrobial agents, and adjunctive therapies for infection control. Future research should focus on elucidating the specific mechanisms of action, optimizing extraction methods for maximum effectiveness, and conducting clinical trials to validate effectiveness in different settings. Additionally, exploring the inclusion of hibiscus in health products, such as wound dressings and topical treatments, could open up new avenues for natural health solutions. Overall, hibiscus represents a valuable opportunity to advance antimicrobial strategies in a world increasingly wary of antibiotic resistance.

## Introduction and background

Due to the emergence of multidrug-resistant pathogens and the resurgence of infectious diseases worldwide, the development of novel antimicrobial agents is urgently needed [1].

In this context, the potential therapeutic properties of natural plant products with some antimicrobial activity have attracted considerable attention. Various species of the genus Hibiscus have been the subject of extensive research due to their potential antimicrobial properties and useful bioactive compounds [2].

With more than 200 different species, Hibiscus is a well-known genus in the Malvaceae family of flowering plants, valued both for its remarkable aesthetics and for its important cultural and medicinal uses [3]. The genus Hibiscus reflects the rich biodiversity of tropical and subtropical regions. Notable members of the genus include *Hibiscus rosa-sinensis (H. rosa-sinensis)*, *H. acetosella*, and especially *H. sabdariffa*, which has attracted widespread attention for its culinary and health benefits, including their antioxidant, anti-inflammatory, and antimicrobial properties [4].

The antimicrobial activity of Hibiscus species is largely due to their diverse phytochemical profiles, which include flavonoids, phenolic acids, anthocyanins, and organic acids. The presence of these bioactive compounds is believed to be related to the plant's effectiveness in inhibiting the growth of several microorganisms, including *Staphylococcus aureus* (*S. aureus*)*, Escherichia coli *(*E. coli*),* Streptococcus* mutans (*St. mutans*), *Enterococcus faecalis *(*E. faecalis*), *Klebsiella pneumonia *(*K. pneumonia*),* *and Candida albicans (*C. albicans*) [5].

The methodology for this review included a thorough literature search across several electronic databases, such as PubMed, Scopus, and Google Scholar. We utilized keywords like "Hibiscus antimicrobial properties", "Hibiscus phytochemicals", "Natural antimicrobial agents", "Synergistic effects of Hibiscus", "Hibiscus tea and health benefits", "Antimicrobial activity of Hibiscus", and "Phytotherapy in infections" in various combinations to find relevant studies. The search was limited to peer-reviewed original articles, clinical trials, and review papers in English. Studies were chosen based on their relevance to recent developments in the field.

In this review, the botanical classification of Hibiscus is briefly discussed, common species are highlighted with emphasis on *H. sabdariffa*, and the traditional uses of the plant in various cultures are examined. The relevance of these applications is highlighted based on findings from the current medical literature.

This review aims to explore the antimicrobial properties of Hibiscus, with particular emphasis on *H. sabdariffa* and its various phytochemical components, illustrate the mechanisms of action behind it, and explore possible applications.

## Review

Botanical classification

The classification of Hibiscus provides insight into its botanical lineage and ecological significance. This plant belongs to the kingdom Plantae and falls under the classes Angiosperms and Eudicots. It belongs to the order Mallow and the family Malvaceae, the specific genus being Hibiscus. This hierarchical taxonomy highlights the place of Hibiscus within the plant kingdom and highlights its importance in various ecological contexts [3].

Common species of hibiscus

Although there are many notable species in the Hibiscus genus, four are particularly noteworthy because of their importance in medicine, culture, and nutrition. *H. sabdariffa* is possibly the most studied species; it is often recognized by its bright red flowers and calyxes. A tart, refreshing tea brewed from it is enjoyed by people from many different cultures. Known as “Bissap” in West Africa and “Sorrel” in the Caribbean, this fruit is valued both for its delicious taste and its high vitamin and mineral content [6].

*H. rosa-sinensis* is often planted as an ornamental plant and is also known as Chinese Hibiscus or shoe flower. Due to its antibacterial and anti-inflammatory properties, it is used in conventional medicine. Studies show that it is good for treating scalp problems and promoting hair development [7,8].

*H. acetosella*, also known as red sorrel, is valued for its adaptability in the kitchen. Due to their spicy taste, their leaves are often used in salads and drinks, especially in African cuisine. Additionally, the plant has several health benefits, including acting as an antioxidant and supporting digestive health [9].

*H. mutabilis*, often called the “Confederate rose,” is known for its large, colorful flowers that change from white to pink as they mature. Its flowers are used for mild medicinal purposes in some cultures, but they are primarily grown for their aesthetic value [10].

Traditional uses in various cultures

Hibiscus has become an integral part of many social circles around the world and serves as an important part of culinary conventions, therapeutic uses, and ceremonial customs. The conventional use of Hibiscus shows its importance beyond unimportant aesthetics [11].

In various African countries, *H. sabdariffa* is brewed into a popular drink called “Bissap”. This soft drink is valued for its rich taste and health-promoting properties such as its potential to reduce body weight and improve liver health. The calyxes are regularly eaten dried or fresh and contribute a sour flavor to portions of mixed vegetables and sauces in meals. In many communities, Hibiscus flowers are served in soups or as a vegetable, highlighting their nutritional value [6].

In India and other parts of Asia, the flowers of *H. rosa-sinensis* are used in conventional medicines to treat various ailments, including aggravation and hair loss. Extracts from the flowers are used in shampoos and conditioners to promote scalp health. Additionally, Hibiscus tea is known for its invigorating properties and is said to aid in digestion, highlighting its role in Indian traditional teas [7].

In the Caribbean, *H. sabdariffa* can be a staple at merry events, particularly known as “Tawny” at Christmas celebrations. This drink, often flavored with ginger and cloves, reflects the social combination of tastes and serves as an image of the neighborhood. The plant is also valued for its high vitamin content including vitamins A, C, and E, which are present in varying but significant amounts. In Middle Eastern countries, Hibiscus tea is known for its sour taste and beneficial effects. It is often served chilled in hot climates as an invigorating refreshment and is often associated with other therapeutic uses including gastric support and antioxidant protection [6].

In North America, while Hibiscus is valued primarily for its decorative qualities in scenes, its potential wellness benefits have received attention beyond that. Hibiscus tea, particularly from *H. sabdariffa*, is increasingly being consumed for its tart taste and potential cardiovascular benefits. Additionally, an ongoing study is examining its effects on metabolic diseases such as obesity and diabetes [12].

Phytochemical components

Hibiscus has a variety of bioactive compounds in its chemical composition, all of which contribute to its nutritional value and therapeutic potential. The richness of organic acids, vitamins, minerals, and polyphenols underlines the importance of Hibiscus in both conventional and alternative medicine [2].

The phytochemical profile of Hibiscus is covered in detail in this review, along with insights into how these elements work together to enhance antimicrobial effectiveness.

Phenolic compounds, which include flavonoids and related structures, are the most abundant group of compounds in Hibiscus. The red color of Hibiscus is due in particular to its high anthocyanin content, especially delphinidin and cyanidin [13]. Depending on the environment and extraction technique, the concentrations of these compounds can change [11]. Studies have shown that anthocyanins are powerful antioxidants that can both reduce inflammation and reduce oxidative stress. Other health-promoting properties of Hibiscus include anti-inflammatory, anti-diabetic, and antimicrobial flavonoids such as quercetin and kaempferol. A study by Touba et al. showed that Hibiscus extracts have potent antimicrobial effects against a range of pathogens, supporting the potential of flavonoid compounds as a therapeutic agent [14].

Ascorbic acid, malic acid, and citric acid are among the organic acids found in *H. sabdariffa*. A study has shown that ascorbic acid, also called vitamin C, is a powerful antioxidant and an essential component of the immune system [6]. These organic acids improve the overall nutritional profile of Hibiscus drinks and contribute to their tangy taste. A study has shown that the organic acids contained in hibiscus can help lower blood pressure and regulate blood sugar [12]. In addition to phytochemicals, hibiscus is a rich source of vitamins and minerals. It contains a variety of nutrients, including calcium, iron, magnesium, and vitamins A and B (thiamine, riboflavin), which are necessary for many body processes such as energy metabolism and bone health [15].

Hibiscus has been found to contain essential oils and terpenes such as limonene and linalool, although in smaller amounts. These substances have aromatic properties and contribute to the taste and scent of goods made with Hibiscus [16]. 

Methods used to extract Hibiscus active compounds and their impact on efficacy

The selection of extraction technique is critical in determining the quantity, quality, and potency of Hibiscus extracts. Methods such as maceration, Soxhlet extraction, ultrasound-assisted extraction (UAE), microwave-assisted extraction (MAE), and supercritical fluid extraction (SFE) have different advantages and disadvantages that affect how well the bioactive compounds are extracted. There is still a great opportunity to use these techniques for food preservation and medicinal purposes as research continues into the most effective methods for extracting Hibiscus compounds. This section provides an overview of the most used extraction techniques and their impact on the potency of the extracted compounds [17].

One of the simplest and most popular extraction methods is maceration, which involves soaking plant materials in a solvent at room temperature for an extended period. This technique allows soluble compounds to gradually diffuse into the solvent. Selecting a solvent is crucial. Water and ethanol are often chosen because they are good at extracting polar compounds such as phenolic acids and flavonoids. Research shows that maceration produces a significant number of bioactive compounds, making it a good technique for producing extracts from Hibiscus flowers. However, longer extraction times can cause sensitive compounds to break down and lose some of their antimicrobial activity. A more effective method that allows continuous extraction from solid plant materials is Soxhlet extraction. The solvent is cyclically condensed in a vertical column and repeatedly seeps through the plant material. Hibiscus has produced high concentrations of flavonoids and anthocyanins with this method and has proven useful for the extraction of non-polar compounds. However, longer times and higher temperatures are often required for Soxhlet extraction; in this process, heat-sensitive compounds degrade and reduce the overall bioactivity of the extract, which can cause problems [18].

Ultrasonic waves improve the penetration of solvents into plant cells. Ultrasound-assisted extraction (UAE) improves compound solubility and extraction efficiency. The UAE has attracted attention due to its ability to produce large yields of bioactive compounds faster than traditional techniques. According to studies, UAE can significantly increase the concentrations of phenolic compounds in Hibiscus while maintaining their antioxidant activity. This approach is promising for the future, as it not only increases the extraction efficiency but also increases the antimicrobial effectiveness of the extracted compounds [19].

By heating the solvent and plant material with microwave energy, MAE accelerates the extraction due to the enhanced diffusion of the compounds. Studies have shown that MAE can extract more flavonoids and anthocyanins from Hibiscus compared to traditional methods. Additionally, MAE is associated with shorter extraction times and solvent consumption, resulting in a more environmentally friendly method. This method is particularly attractive for pharmaceutical and food preservation applications because the higher temperature and pressure during the extraction process often improve the antimicrobial properties of the resulting extracts [20].

Supercritical carbon dioxide is the solvent used in SFE, which extracts bioactive compounds at high pressures and temperatures. This process produces highly pure extracts with little solvent residue and has a high selectivity for non-polar compounds. Due to its cost and complexity, SFE is less commonly used for Hibiscus extraction; however, it has shown promise in producing high-quality extracts with strong antimicrobial properties [21].

Antimicrobial properties of Hibiscus extracts

In conventional medicine, Hibiscus has long been used primarily to treat inflammatory and infectious diseases. Hibiscus extracts have been shown in numerous studies to have antimicrobial properties against a variety of pathogens such as viruses, fungi, and bacteria. Venkatesan et al. investigated the antimicrobial activity of *H. sabdariffa* extract against a variety of pathogens, including *S. aureus*, *E. coli*, *Pseudomonas aeruginosa,* and *C. albicans*. The results of the study showed the strong antimicrobial activity of the extract against *S. aureus* and *E. coli*. The study shows that the antimicrobial activity of the crude metabolites of *H. sabdariffa* has a 13 mm zone of inhibition for *S. aureus*, followed by an 11 mm zone of inhibition for *E. coli* at a concentration of 5 mg/ml. The minimum inhibitory concentration (MIC) for *S. aureus* is 256 and for *E. coli*, the value is 512 [22].

Other studies also showed Hibiscus extracts have also shown antifungal and antiviral activity [14,23]. These results highlight the potential of Hibiscus as a naturally occurring antimicrobial agent that can be used for a variety of purposes.

When it comes to Hibiscus extracts, two of the most researched bacterial pathogens are *S. aureus* and Gram-negative bacteria such as *E. coli*, which are often associated with foodborne illnesses while Gram-positive bacteria, such as *S. aureus*, are known to be involved in a variety of human infections, ranging from skin infections to more serious illnesses such as sepsis and pneumonia.

Enteropathogenic* E. coli* (EPEC) is a leading cause of food and water-borne diarrheal disease worldwide, particularly in developing countries. The MIC and minimum bactericidal concentration (MBC) of *H. sabdariffa* extract against EPEC were determined in vitro using the broth microdilution test to evaluate the antibacterial activity of the aqueous extracts against EPEC and the control strain of *E. coli*. The MIC values for EPEC and *E. coli* were 6.5 mg/ml and mg/ml against the control strain. Since occasional growth was observed at 20 mg/mL, the MBC of the HS extract versus EPEC was calculated to be 25 mg/ml [24].

Abass et al. investigated how an aqueous extract of *H. Sabdariffa* had an antibacterial effect. Metronidazole, tetracycline, amoxicillin-clavulanic acid, and chlorhexidine had an effect on the bacteria *St. mutans*, *S. aureus,* and *E. faecalis*. The antimicrobial properties of *H. sabdariffa* at concentrations of 50 mg/ml and 100 mg/ml were equivalent to those at concentrations of 0.2% and 2% chlorhexidine, respectively. In terms of antibacterial activity against *St. mutans*, *S. aureus*, and *E. faecalis*, metronidazole was less effective than *H. sabdariffa* at 75 and 100 mg/ml. They were superior to tetracycline with regard to *E. faecalis*. The MIC for *E. faecalis* could be as low as 16 mg/ml [25].

Another study observed the antimicrobial activity of* H. sabdariffa* extract against uropathogenic strains isolated from recurrent urinary tract infections. The MIC of the extract varied between 0 and 4 mg/ml for all uropathogenic *E. coli* and *Klebsiella pneumonia* isolates while MBC values ​​varied between 8 and 64 mg/ml. Both the MBC-MIC ratio and the time-kill experiment demonstrated the overall bacteriostatic effect of the extracts. The results of the biofilm capacity inhibition test showed that the extracts prevented all isolates from producing biofilm. However, the degree of biofilm inhibition of the bacterial strains varied, ranging from 8% to 60% reduction in optical density [26].

Hibiscus extracts can sometimes be more effective when used in conjunction with other antimicrobial agents. Hassan et al. studied the interactions between Hibiscus extract and standard antibiotics for treating *Helicobacter pylori *and found that the combination has an additive effect and was obtained against five of seven tested strains [27].

The antifungal effect was attributed to the destruction of the fungal cell wall by phytochemicals, highlighting the need for natural substitutes, particularly for the treatment of opportunistic fungal infections. Scientists examined the effectiveness of several *H. sabdariffa* extracts as antifungal agents against different strains of Candida species. One thousand (1000) mg/ml of the extract shows the highest inhibition zone against *C. albicans*, *C. tropicalis,* and *C. glabrata*, which are 9 mm, 8 mm, and 9 mm, respectively. It was found that ethanol extract of *H. rosa-sinensis* showed more potent anti-candidiasis compared with methanol extract [28].

Fungal pathogens pose a serious threat to food safety and clinical settings. Hibiscus extracts have been shown to possess antifungal properties in numerous studies. A recent study demonstrated the potential of Hibiscus extracts in the treatment of fungal infections, as a 10% Hibiscus extract achieved a MIC of 2 mg/ml against *C. albicans* [14].

Certain enzymes that are important for the growth and metabolism of microorganisms can be inhibited by the phenolic compounds contained in Hibiscus. Hibiscus extracts can prevent the growth and survival of microorganisms by interfering with their enzymatic functions [29].

Modulation of Biofilm Formation

Based on certain research, Hibiscus extracts can prevent the formation of biofilms by bacteria. The ability of Hibiscus to prevent the formation of biofilms, which are complex communities of microorganisms resistant to antibiotic treatment, makes it a potentially effective weapon in the fight against antibiotic resistance [30].

Induction of Oxidative Stress

The bioactive compounds of Hibiscus have the potential to damage the DNA, proteins, and lipids of microbial cells by inducing oxidative stress. The antioxidant defenses of microbial cells could be overwhelmed by this oxidative damage, leading to their demise or reduced viability [30].

The antifungal activities of *H. sabdariffa* extract provide fundamental evidence for the potential effects of this plant in preventing recurrent candiduria caused by* C. albicans* in combination with conventional antifungal agents. Different levels of MIC of the extract were examined against fluconazole. Resistant *C. albicans* isolated from recurrent candiduria were observed in all isolates. The MIC values ​​were between 0.5 and 2.0 mg/ml. The time-killing experiment showed that the effect was fungistatic. The results of the biofilm inhibition test showed that *H. sabdariffa* extract inhibited the biofilm production of all isolates [31,32].

The potential of Hibiscus as a natural preservative in the food industry is highlighted by the antimicrobial properties of Hibiscus extracts and their particular MIC values. Hibiscus extracts may improve food safety and promote health in food formulations, which is in line with consumers' growing preference for natural and clean-label products. Because of the strong MIC values shown, preliminary research has successfully applied Hibiscus extracts to prevent microbial spoiling in a variety of food products, including dairy, meat, and beverages [33].

Studies have shown that Hibiscus extracts can help extend the shelf life of various foods, such as sauces and juices, by blocking the growth of spoilage microorganisms [34].

The oral health benefits of Hibiscus are another exciting area of ​​research. The antimicrobial properties of Hibiscus extract were studied in patients with periodontitis. The results of the randomized controlled trial showed that the Hibiscus extract mouthwash group significantly reduced the number of bacteria in their oral cavity compared to the placebo group. The results suggest that Hibiscus is a potential adjunctive treatment for preventing periodontal problems and improving oral health [35].

When Hibiscus is mixed with other natural extracts or traditional antimicrobial agents, synergistic effects can occur, which have been studied in clinical studies. Olusanya et al. found that the antibacterial activity of Hibiscus extract increased when garlic extract and Hibiscus extract were combined to treat *S. aureus*. This highlights how Hibiscus can be used in combination therapies through integrative approaches. Hibiscus also has anti-inflammatory properties. Symptoms and complications are often caused by infections, which often produce an inflammatory response [36].

Mechanisms of action of antimicrobial activity of Hibiscus extracts

**Figure 1:**
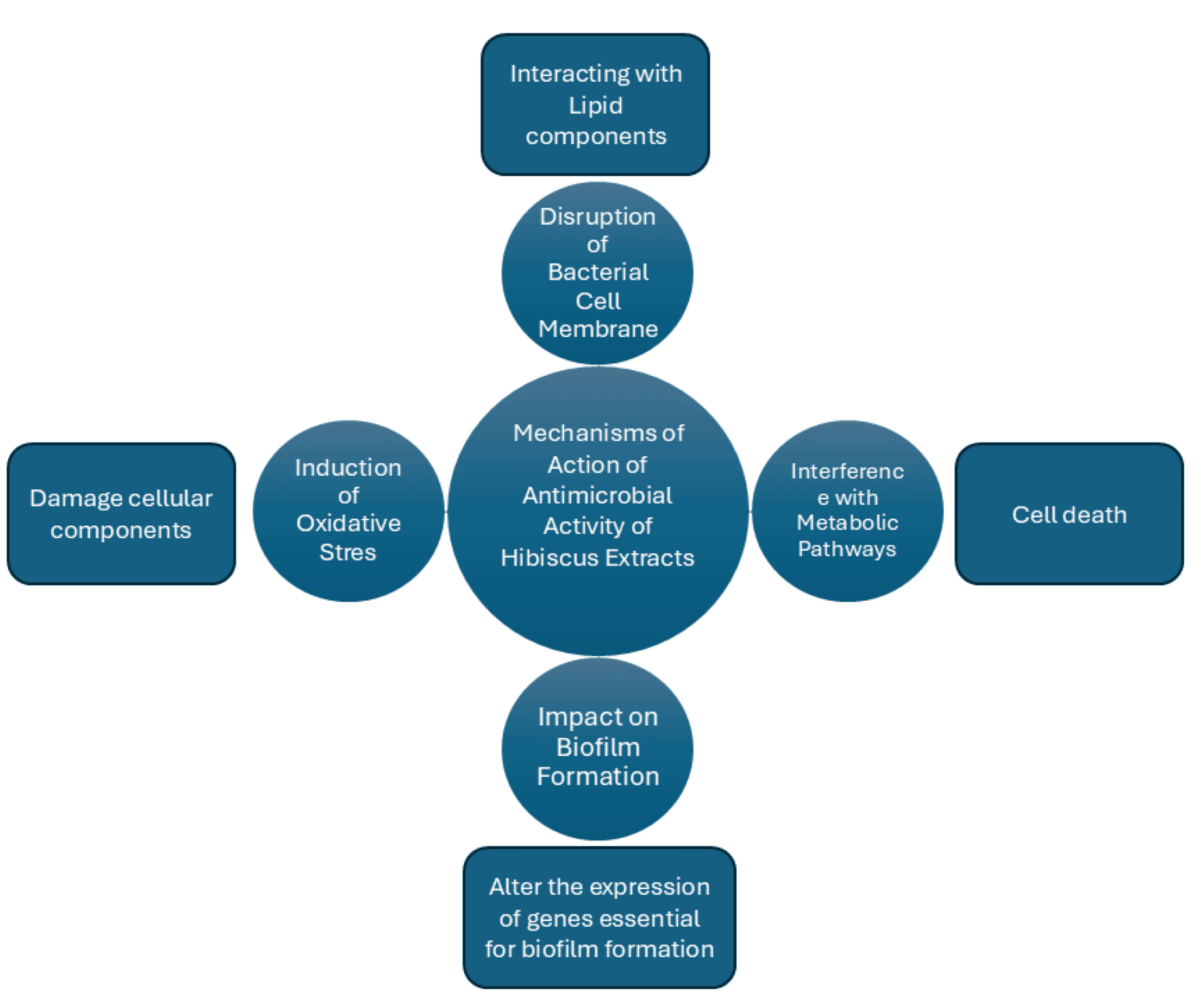
Different mechanisms of antimicrobial action of Hibiscus extracts

Hibiscus extracts have antimicrobial effects through a variety of mechanisms that involve complex interactions between microbial cells and phytochemicals found in the plant (Figure 1). Acquiring knowledge of these mechanisms can aid in their application in food preservation and medicine.

Disruption of Bacterial Cell Membrane

The breakdown of microbial cell membranes is one of the main ways in which Hibiscus extracts exert their antibacterial effects. Hibiscus phytochemicals have the ability to alter the permeability and structure of microbial membranes by interacting with their lipid components. This disruption causes intracellular contents to leak, which in turn leads to cell death. A study found that the hydrophobic properties of flavonoids from Hibiscus allow them to penetrate lipid bilayers and disrupt the membrane integrity of pathogens such as *S. aureus* and *E. coli*. This interaction not only destroys the membrane but also hinders critical functions necessary for microbe survival such as waste excretion and nutrient absorption [37].

Interference With Metabolic Pathways

The antibacterial properties of Hibiscus extracts are further enhanced by evidence that they inhibit certain enzymatic activities in microbes. The phenolic compounds of Hibiscus have the ability to block microbial enzymes that are essential for metabolic processes such as ATP synthesis and glycolysis. For example, studies show that certain bacterial hydrolases and dehydrogenases can be inhibited by Hibiscus extracts. This inhibition impairs metabolism and energy production, leading to stunted growth and increased susceptibility to environmental stressors. Hibiscus extracts specifically undermine the metabolic potential of pathogens by targeting these vital enzymes, enabling their elimination [38].

Induction of Oxidative Stress

The production of reactive oxygen species is another way in which Hibiscus exerts its antimicrobial activities (ROS). Oxidative stress may be caused by the antioxidant properties of Hibiscus phytochemicals, which can increase ROS production in microbial cells. Cell death may result from damage to proteins, lipids, and DNA, as well as other cellular components, from this stress. Another study showed that Hibiscus extracts can increase the production of reactive oxygen species (ROS) in certain bacterial strains, especially when they are stressed. An important mechanism for the antimicrobial effect of Hibiscus was impaired microbial viability [39].

Impact on Biofilm Formation

Microbial communities form biofilms, which are protective structures that provide resistance to antimicrobial agents and make it difficult to treat infections. According to recent research, Hibiscus extracts can prevent the formation of biofilms in a variety of pathogenic bacteria such as *P. aeruginosa* and *St. mutans*. The mechanism of this activity is likely related to the ability of Hibiscus compounds to alter the expression of genes essential for biofilm formation. For example, flavonoids and phenolic compounds have the ability to intervene in signaling pathways that control the formation of biofilms, preventing bacteria from adhering to and accumulating on surfaces [40].

## Conclusions

With increasing antibiotic resistance, the antimicrobial properties of Hibiscus species, particularly *H. sabdariffa*, have recently attracted great interest. Significant antibacterial, antifungal, and antiviral properties of Hibiscus extracts have been demonstrated in numerous studies against a variety of pathogens, including Gram-positive and Gram-negative bacteria. Antibiotic resistance (AMR) poses a growing threat to global health and requires creative solutions that go beyond traditional antibiotic treatments. Incorporating natural products, such as those made from Hibiscus, is a viable approach to overcoming this obstacle. Natural substances often have different mechanisms of action that are less likely to lead to resistance. Researchers and medical professionals can develop innovative formulations that not only increase the effectiveness of current antibiotics but also reduce the need for artificial compounds by harnessing the antimicrobial potential of Hibiscus.

Educating consumers about the safe and efficient use of natural products as complementary therapy in healthcare is extremely beneficial given the growing interest of the general public in herbal remedies. Ultimately, the antimicrobial properties of Hibiscus highlight a critical path forward in the fight against antimicrobial resistance. To ensure that these natural products can be safely and effectively incorporated into therapeutic treatment regimens, it is essential that advances in research continue to elucidate, through clinical studies and trials, the precise mechanisms by which Hibiscus exerts its antimicrobial effects. Novel treatments that take advantage of the complex pharmacological profiles of plants such as Hibiscus may be made possible through continued collaboration between researchers in modern medicine and traditional medicine practitioners.
